# 89 Second-Degree versus Third-Degree Burn Differences in Length of Stay

**DOI:** 10.1093/jbcr/irad045.062

**Published:** 2023-05-15

**Authors:** Vishal Bandaru, Brandon Youssi, Ryan Morgan, Kevin Nguyen, Xiyu Liu, Tristin Chaudhury, Zarif Gani, Chip Shaw, Alan Pang, John Griswold

**Affiliations:** Texas Tech University Health Sciences Center, Lubbock, Texas; Texas Tech University Health Sciences Center, Lubbock, Texas; Texas Tech University Health Sciences Center, Lubbock, Texas; Texas Tech University Health Sciences Center, Happy Valley, Oregon; Texas Tech University Health Sciences Center, Lubbock, Texas; Texas Tech University Health Sciences Center, Lubbock, Texas; Texas Tech University Health Sciences Center, Lubbock, Texas; Texas Tech University Health Sciences Center, Lubbock, Texas; Texas Tech University Health Sciences Center, Lubbock, Texas; Texas Tech University Health Sciences Center, Lubbock, Texas; Texas Tech University Health Sciences Center, Lubbock, Texas; Texas Tech University Health Sciences Center, Lubbock, Texas; Texas Tech University Health Sciences Center, Lubbock, Texas; Texas Tech University Health Sciences Center, Happy Valley, Oregon; Texas Tech University Health Sciences Center, Lubbock, Texas; Texas Tech University Health Sciences Center, Lubbock, Texas; Texas Tech University Health Sciences Center, Lubbock, Texas; Texas Tech University Health Sciences Center, Lubbock, Texas; Texas Tech University Health Sciences Center, Lubbock, Texas; Texas Tech University Health Sciences Center, Lubbock, Texas; Texas Tech University Health Sciences Center, Lubbock, Texas; Texas Tech University Health Sciences Center, Lubbock, Texas; Texas Tech University Health Sciences Center, Lubbock, Texas; Texas Tech University Health Sciences Center, Happy Valley, Oregon; Texas Tech University Health Sciences Center, Lubbock, Texas; Texas Tech University Health Sciences Center, Lubbock, Texas; Texas Tech University Health Sciences Center, Lubbock, Texas; Texas Tech University Health Sciences Center, Lubbock, Texas; Texas Tech University Health Sciences Center, Lubbock, Texas; Texas Tech University Health Sciences Center, Lubbock, Texas

## Abstract

**Introduction:**

Length of stay (LOS) is a crucial element to patient care plans, hospital profit, and a patient’s well-being. However, predicting LOS is difficult as burn injuries often have complications. The expectation for LOS in the burn ward is generally one day per percent total body surface area (TBSA) burned. This prediction approximates the average but rarely represents true patient LOS. Healing times for second and third-degree burns differ, yet most studies only evaluate TBSA rather than 2^nd^ and 3^rd^ degree burns separately. We hypothesized that third-degree burns would have a longer LOS than same sized second-degree burns.

**Methods:**

We obtained a list of all patients diagnosed with Second/Third-degree and verified the inclusion of patients meeting the study criteria from July 01, 2011, to July 01, 2021. Afterward, data was manually extracted from electronic health records and separated from patients with inhalation injury. We placed the aggregate findings in a 3D scatter plot to develop a predictive LOS formula.

**Results:**

The initial data set (n=678) decreased due to missing burn documentation and exclusion of the inhalation injury group yielding the final data set (n=388). For every one percent second-degree burn increase, the LOS (in days) increases by 0.587 + 0.057 (p= 3.07E-22). For every third-degree burn percent increase, LOS increases by 1.328 + 0.086 (p=1.4E-42). The intercept for the 3D scatter plot was set to 0 as a patient without burns or inhalation injury should have a LOS of 0. The r-squared value is 0.58 which shows a medium level of correlation. Our current equation – LOS = 0.587(2^nd^ degree-burn) +1.328(3^rd^ degree-burn).

**Conclusions:**

The one day per percent TBSA rule does not accurately predict LOS. Third-degree burn percentage may double the length of stay when compared to second-degree burns. More closely following 2^nd^ v. 3^rd^ degree burn and other comorbidities will give patients a more accurate estimate of their LOS.

**Applicability of Research to Practice:**

Insurance, physicians, and patients are all affected by LOS. Insurance companies require accurate depictions of LOS to ensure sufficient funding for their patients’ hospital stay. Physicians use LOS to assess course of healing and if other problems are hindering recovery. Furthermore, hospital stay changes a patient’s daily life, work, education, and family. Better predictions for LOS may allow patients to fully grasp what to expect with their recovery.

